# Supplementary material for: Identification and Characterization of VDAC Family in Maize
Source: Plants (Basel). 2023 Jul 4;12(13):2542. doi: 10.3390/plants12132542 (PMC10346152; doi:10.3390/plants12132542)
Supplement: Supplementary file 1 [file plants-12-02542-s001.zip › plants-2460448-supplementary.pdf]

Identification and characterization of VDAC family in maize

Carolina Rodríguez-Saavedra[1], Donají Azucena García-Ortiz[1], Andrés Burgos-Palacios[1], Luis Enrique Morgado-Martínez[1], Beatriz King-Díaz[1], Arturo Guevara-García[2] and Sobeida Sánchez-Nieto[1]*

[1]Laboratorio de Transporte y Percepción de Azúcares en Plantas, Departamento de Bioquímica, Facultad de Química, Universidad Nacional Autónoma de México, Mexico City, Mexico; carol_unam10@hotmail.com (C.R.-S.); 418014145@quimica.unam.mx (D.A.G.-O.); aburgosbp@gmail.com (A.B.-P.); enrque.mgdomtz@gmail.com (L.E.M.-M.); kingbeat@unam.mx (B.K.-D.)

[2]Departamento de Biología Molecular de Plantas, Instituto de Biotecnología, Universidad Nacional Autónoma de México, Cuernavaca, México; arturo.guevara@ibt.unam.mx

***Correspondence**: sobeida@unam.mx

SUPPLEMENTARY MATERIAL

Table S1. Maize VDAC sequences found on Ensembl Plants, NCBI, Maize GDB, ENA and UniProt databases

#	Name	Chromosomic localization	Nucleotide sequence length (bp)	Amino acid sequence	Molecular weight (kDa)	ID UniProt
**1**	ZmVDAC1a	chr 2: 181,511,123-181,512,600	831	276	29.48	Q9SPD9
**2**	ZmVDAC1b	chr7: 102,142,586-102,146,487	831	276	29.65	A0A1D6I2X6
**3**	ZmVDAC2_2	chr6:172,936,942-172,940,416	831	276	29.18	Q9SPD7
**4**	ZmPorin1_0	chr8: 143,733,235-143,739,212	828	275	29.78	K7VJ77
**5**	ZmVDAC3_1	chr3: 225,067,705-225,070,108	828	275	29.79	B4FX24
**6**	ZmVDAC4	chr9: 158,366,536-158,370,125	831	276	29.59	B6T1E3
**7**	ZmOMMPP	chr1: 23,921,332-23,928,650	828	275	29.23	A0A1D6JS28
**8**	ZmMOMPP2	chr9:155,580,106-155,585,227	828	275	29.18	C4IYM7
**9**	ZmUP	chr1: 8,367,595-8,373,479	831	276	29.62	A0A804LDA0
**10**	ZmOPMPPPOR1	chr8: 143,839,123-143,845,017	834	277	29.98	P42057
**11**	ZmOMM	chr3: 208,258,352-208,260,807	834	277	29.91	B6TAU9
**12**	ZmMOMPP4	chr1: 8,304,556-8,306,909	852	283	30.43	A0A1D6JN64
**13**	ZmVDAC3_0	chr8: 139,640,236-139,648,936	834	277	29.37	A0A1D6FWS5
**14**	ZmVDAC2_0	chr6: 160,830,973-160,834,492	915	304	32.41	A0A3L6EA00
**15**	ZmVDAC2_1	chr8: 139,383,756-139,390,389	945	314	33.29	A0A3L6DRN5
**16**	ZmPorin1_1	chr8: 143,840,897-143,843,253	912	303	32.93	A0A1D6FYA4
**17**	ZmOMMPorin1	chr1: 8,298,475-8,301,181	768	255	27.39	A0A1D6JN63
**18**	ZmVDAC6	chr1: 9,306,992-9,316,363	1275	424	45.00	A0A317Y8M0

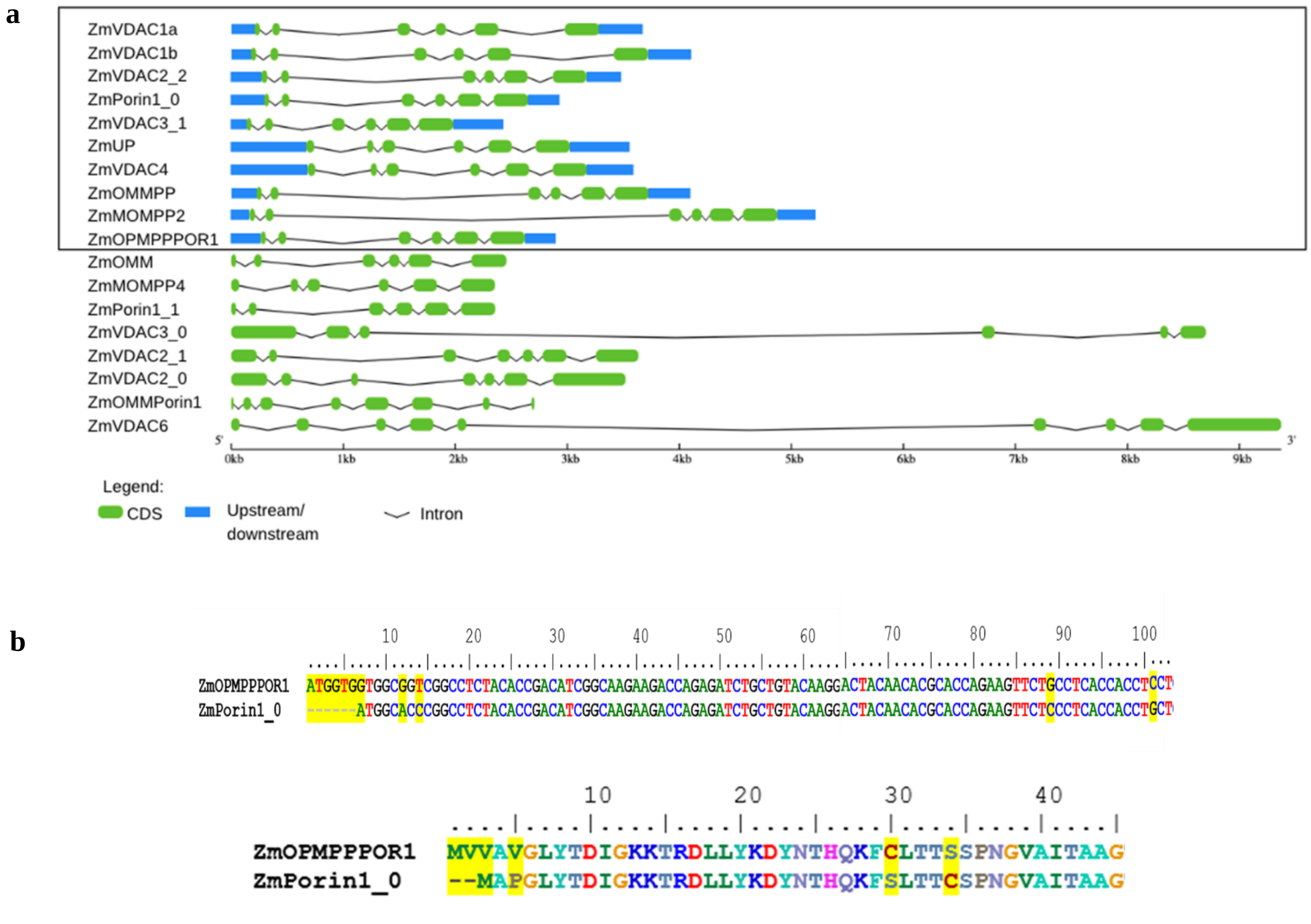

Figure S1. Gene structure of each *ZmVDAC* family member and comparison of two partial sequences. (**a**) *ZmVDAC* genes structures. Non-coding regions are shown in black lines, exons in green boxes and the UTR regions in blue rectangles. The first 10 sequences (inside of the rectangle) contain both UTR regions. The rest of the sequences do not contain both UTR 5' and 3' regions. (**b**) Alignment of the 5´end of the nucleotide sequences of ZmOPMPPPOR1 and ZmPorin1_0 and comparison of the first 40 amino acids of both sequences. The 6 nucleotides observed in ZmOPMPPPOR1, a double repeat of GTG, produced an additional 2 amino acids that are not found in ZmPorin1_0 or in any other ZmVDAC sequence.

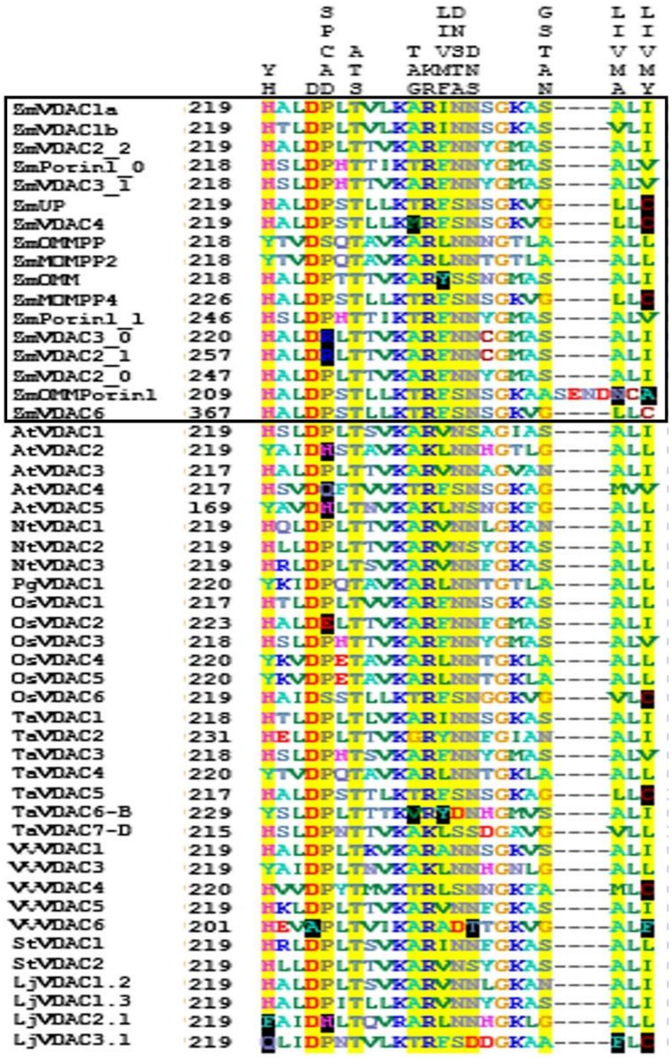

Figure S2. Alignment of plant VDAC orthologues and identification of the MPS domain. ZmVDAC sequences are enclosed by a black rectangle. Amino acids from the conserved and divergent domains are highlighted in yellow and black, respectively. Sequences were taken from UniProt Database: *Arabidopsis thaliana:* AtVDAC1 (Q9SRH5), AtVDAC2 (Q9FJX3), AtVDAC3 (Q9SMX3), AtVDAC4 (Q9FKM2), AtVDAC5 (Q9M2W6) [8]; *Nicotiana tabacum:* NtVDAC1 (A9CM20), NtVDAC2 (A9CM21), NtVDAC3 (A9CM22) [7]; *Pennisetum glaucum*: PgVDAC1 (Q7Y1C6) [27]; *Oryza sativa*: OsVDAC1 (Q6K548), OsVDAC2 (Q6L5I5), OsVDAC3 (Q7F4F8), OsVDAC4 (Q84P97), OsVDAC5 (Q84P97), OsVDAC6 (Q10S27) [27]; *Triticum aestivum*: TaVDAC1 (P46274), TaVDAC2 (A0A3B5Z3Y7), TaVDAC3 (Q41591), TaVDAC4 (W5DXN4), TaVDAC5 (A0A3B6JP43), TaVDAC6-B (A0A3B6FNX1), TaVDAC7-D (A0A3B6TLQ5) [12]; *Vitis vinicola*: VvVDAC1 (A0A438HDA2), VvVDAC3 (A5AQ65), VvVDAC4 (D7SQ96), VvVDAC5 (A5BE54), VvVDAC6 (D7SIG0) [10]; *Solanum tuberosum:* StVDAC1 (P42055), StVDAC2 (P42056) [27]; *Lotus japonicus:* LjVDAC1.2 (Q6W2J4), LjVDAC1.3 (Q6W2J3), LjVDAC2.1 (Q6W2J2) and LjVDAC3.1 (Q6W2J1) [9].

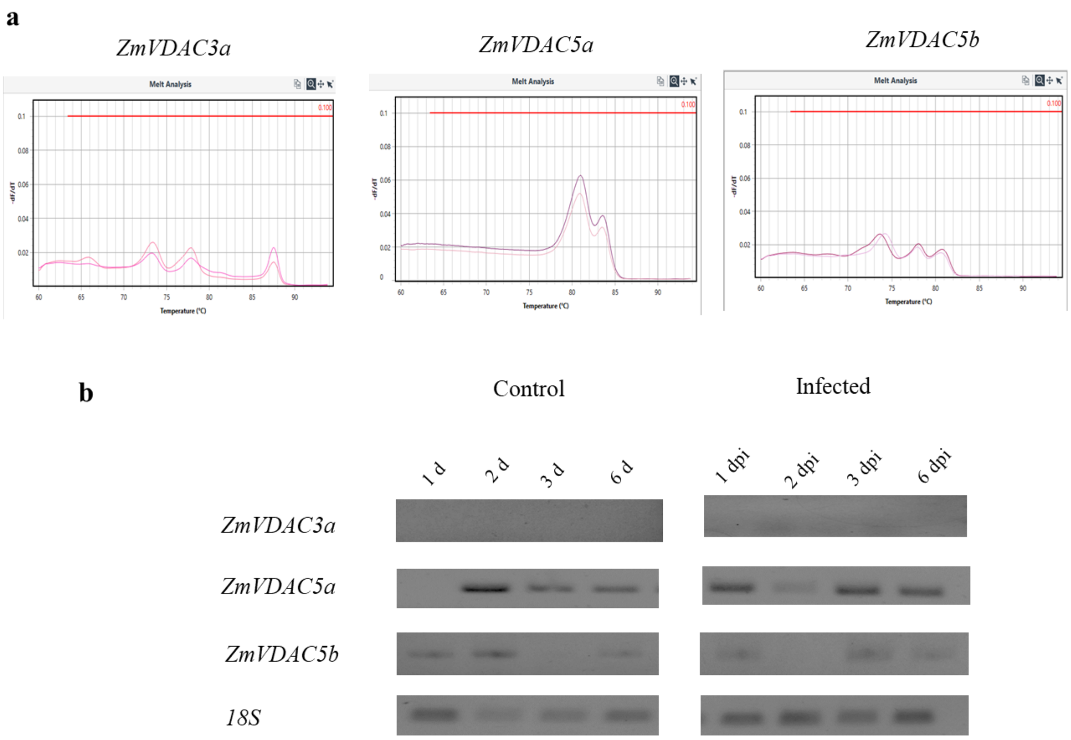

Figure S3. Detection of *ZmVDAC3a*, *ZmVDAC5a* and *ZmVDAC5b*. (**a**) Melting curves of amplified product using qPCR and the designed primers for detect *ZmVDAC3a*, *ZmVDAC5a* and *ZmVDAC5b*, the curves show no specific amplification of the primers. (**b**) Transcript levels of *ZmVDAC3a*, *ZmVDAC5a* and *ZmVDAC5b* during 6 d of germination and in response to *F. verticillioides* infection.

Table S2. Identity matrix of the nine *ZmVDAC* amino acid sequences. Matrix was made using Clustal 2.1 server.

	ZmVDAC1a	ZmVDAC1b	ZmVDAC2	ZmVDAC3a	ZmVDAC3b	ZmVDAC4a	ZmVDAC4b	ZmVDAC5a	ZmVDAC5b
**ZmVDAC1a**	**100**								
**ZmVDAC1b**	91.3	**100**							
**ZmVDAC2**	69.2	67.8	**100**						
**ZmVDAC3a**	65.8	65.8	66.5	**100**					
**ZmVDAC3b**	64.4	63.6	65.1	91.6	**100**				
**ZmVDAC4a**	46.4	47.2	42.9	44.3	44.7	**100**			
**ZmVDAC4b**	46.4	46.4	42.2	43.9	44.7	94.2	**100**		
**ZmVDAC5a**	46.2	46.6	45.6	40.2	39.4	41.1	41.4	**100**	
**ZmVDAC5b**	47.3	47.6	46.3	42.0	40.9	41.1	40.7	96.4	**100**

a

b

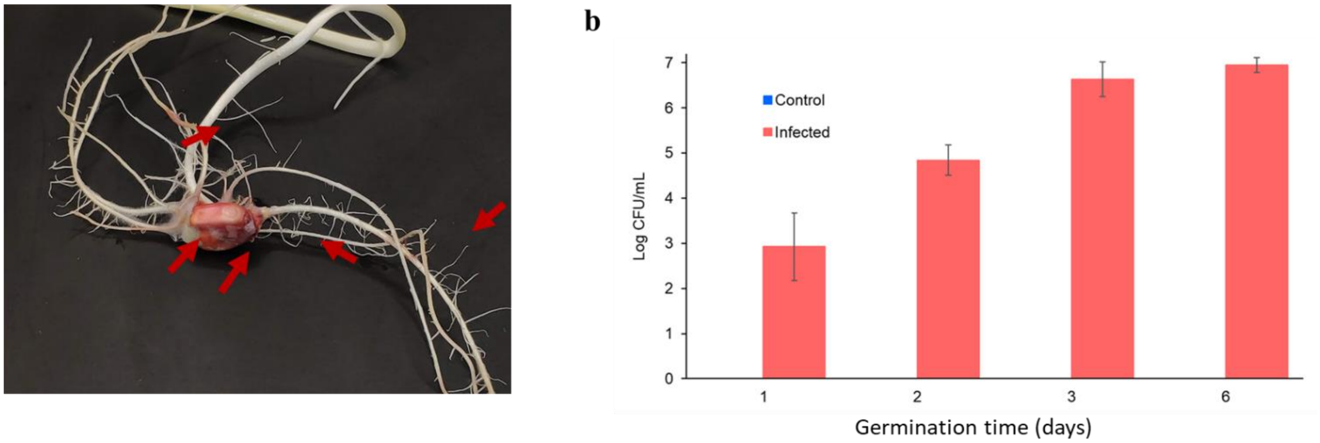

Figure S4. Confirmation of seedling infection by *F. verticillioides* **(a)** Close look of *F. verticillioides* infected seedlings at 6 d of germination. Red arrows show pink pigmentation areas, a characteristic of *Fusarium* growth. (**b)** Proliferation of *F. verticillioides* conidia obtained from the growing control and infected seedling is expressed in Logarithm (10) (Log CFU) during 6 dpi

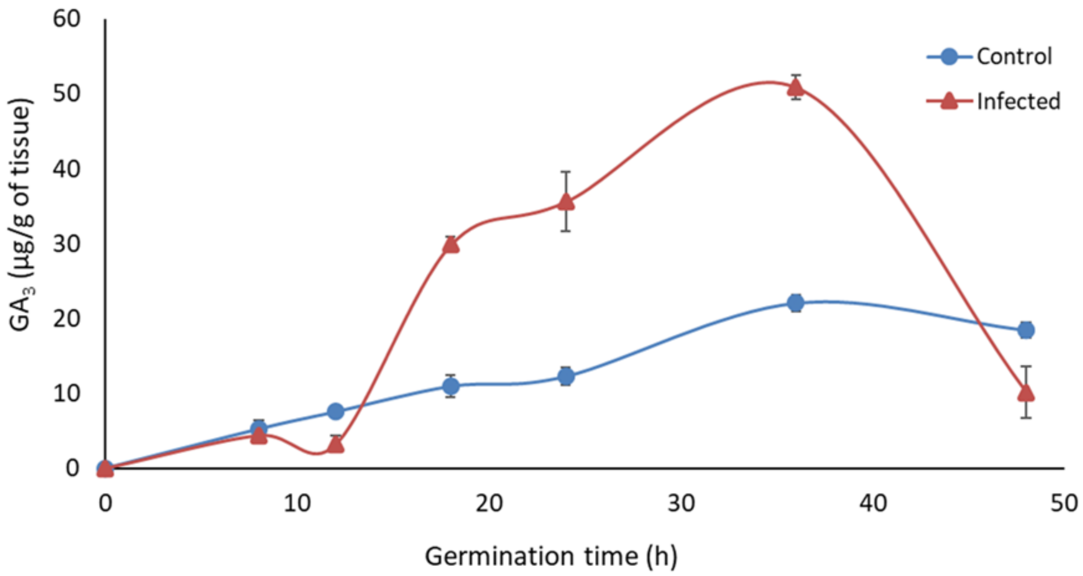

Figure S5. GA3 levels in germinated maize embryos in presence (infected, red) and absence (control, blue) of *F. verticillioides*. Gibberellic acid was extracted from maize embryos and determined by HPLC according to Pan et al., 2008. The samples were injected in an HPLC SHIMADZU® (Analytical and Measuring Instruments Division, Kyoto, Japan) outfitted with UV-visible dual SPD-10A detector, an automatic injector SIL-10AD (VP) and a LC-10AT (VP) pump. The C18 Hibar® 150-4,6 Purospter® STAR/RP-18 was used for the separation. The sample and the standard were separated with acetonitrile:water (24:76) with a constant flux of 1 mL/min. Manipulation and processing of the data were made using LC solution software. The experiment was carried out with three biological samples and each one was determined by triplicate.

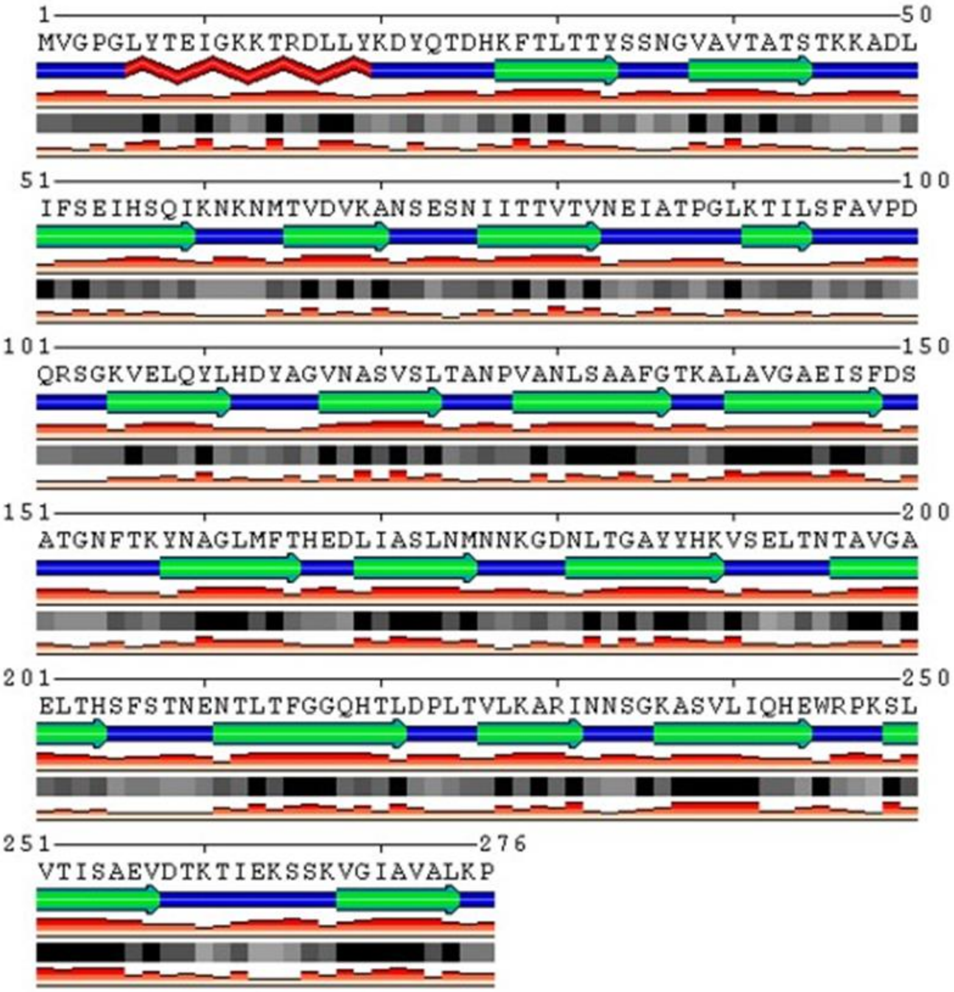

Figure S6. Secondary structure prediction of ZmVDACs amino acid sequences. All 9 sequences analyzed presented 19 β-sheets (green), 21 turns (blue) and a single α-helix at N-terminal region (red). The prediction was performed using MINNOU server. For practicality purposes, only the prediction for ZmVDAC1b is presented.

Table S3. Semi-quantitative RT-PCR conditions for amplification of *ZmVDAC3a*, *ZmVDAC5a*, *ZmVDAC5b* and *Zm18S*.

Gene	Stage		Temperature	Time
ZmDAC3a	Pre-denaturation		94° C	5 min
	Denaturation	30 cycles	94° C	40 s
	Annealing		54° C	1 min
	Extension		72°C	40 s
	Final extension		72° C	7 min
ZmVDAC5a	Pre-denaturation		94° C	5 min
	Denaturation	30 cycles	94° C	40 s
	Annealing		55° C	40 s
	Extension		72° C	40 s
	Final extension		72° C	7 min
ZmVDAC5b	Pre-denaturation		95° C	5 min
	Denaturation	30 cycles	95° C	40 s
	Annealing		50° C	40 s
	Extension		72° C	40 s
	Final extension		72° C	5 min
Zm18S	Pre-denaturation		94° C	5 min
	Denaturation	30 cycles	94° C	40 s
	Annealing		54° C	1 min
	Extension		72° C	40 s
	Final extension		72° C	7 min

Table S4. Primers used to detect *ZmVDAC1a, ZmVDAC2, ZmVDAC3a, ZmVDAC3b, ZmVDAC4a, ZmVDAC4b*, *ZmVDAC5a*, *ZmVDAC5b*, *Zm18S*, *ZmPR1*, *ZmPDF.1 and ZmMCAS6* genes transcript levels by RT-PCR and qPCR.

Gene	Primer sequence	Product length (bp)	Efficiency values (%)
ZmVDAC1a	Fw5'-GAAAGCTGACCTGATCCTTG-3'	244	90.0
	Rv5'-AGGATTGGCTGTCAGACCG-3'		
ZmVDAC1b	Fw5'-AGGGATCTGCTGTACAAGGAC-3'	134	89.8
	Rv5'-ATCTGTGAATGGATCTCACTG-3'		
ZmVDAC2	Fw5'-ACAGCTCAGTCCTCTTCAAC-3'	141	93.5
	Rv5'-CTTCCCGATCTCGGAGTAGA-3'		
ZmVDAC3a	Fw5'-GAGACACGATGTGATATTCCTG-3'	191	NS [a]
	Rv5'-TGGTCTTCTTGCCGATGTC-3'		
ZmVDAC3b	Fw5'-CTACACCGACATCGGCAA-3'	145	91.5
	Rv5'-ATCGCCAAAGGTGGACTC-3'		
ZmVDAC4a	Fw5'-CTAAAGTGTCAACCACAGTCAC-3'	216	92.2
	Rv5'-CGAACTCAGCACCAATGGC-5'		
ZmVDAC4b	Fw5'-GCTTAGCGGCTCTGGTTTGAATCTCA-3'	113	91.6
	Rv5'-CGGTGACATCAATAGTAGTTC-3'		
ZmVDAC5a	Fw5'-GATCCTGGTGAATTCGACAC-3'	214	NS [a]
	Rv5'-ATCCGGATCTAAGCCACAG-3´		
ZmVDAC5b	Fw5'-CTGATCAGATCATCCCATGTTG-3'	206	NS [a]
	Rv5'-ATTTACCAACGGCCAGATG-3'		
Zm18S [65]	Fw5'-CCATCCCTCCGTAGTTAGCTTCT	150	93.3
	Rv5'-CCTGTCGGCCAAGGCTATATAC		
ZmPR1	Fw5'-GGCCACTACACCCAGATCAT-3'	200	86.5
	Rv5'-TGGGACAGCAAGAGACACAG-3'		
ZmPDF.1	Fw5'-GCTTCAAGGGACCTTGCTC-3'	337	87.6
	Rv5'-AAACGCGCCATAGAGTTCAT-3'		
ZmMCAS6	Fw5'- CTCGTCATTCTGGTTCTG-3'	214	89.6
	Rv5'- CCAGTTTGTTCCTTGAGC-3'		

[a] NS – Not specific for qPCR assay.

Supplementary references

Clustal Omega. Multiple Sequence Alignment. Available online : https://www.ebi.ac.uk/Tools/msa/clustalo/ (accessed on 25 May 2023)

Pan, X., Welti, R., Wang, X. Simultaneous quantification of major phytohormones and related compounds in crude plant extracts by liquid chromatography–electrospray tandem mass spectrometry. *Phytochemistry* 2008, 69 (8), 1773-1781. https://doi.org/10.1016/j.phytochem.2008.02.008.
